# Supplementary material for: Flexible graphene-based neurotechnology for high-precision deep brain mapping and neuromodulation in Parkinsonian rats
Source: Nat Commun. 2025 Mar 25;16:2891. doi: 10.1038/s41467-025-58156-z (PMC11937542; doi:10.1038/s41467-025-58156-z)
Supplement: Supplementary file 1 — Supplementary Information [file 41467_2025_58156_MOESM1_ESM.pdf]

Supplementary information

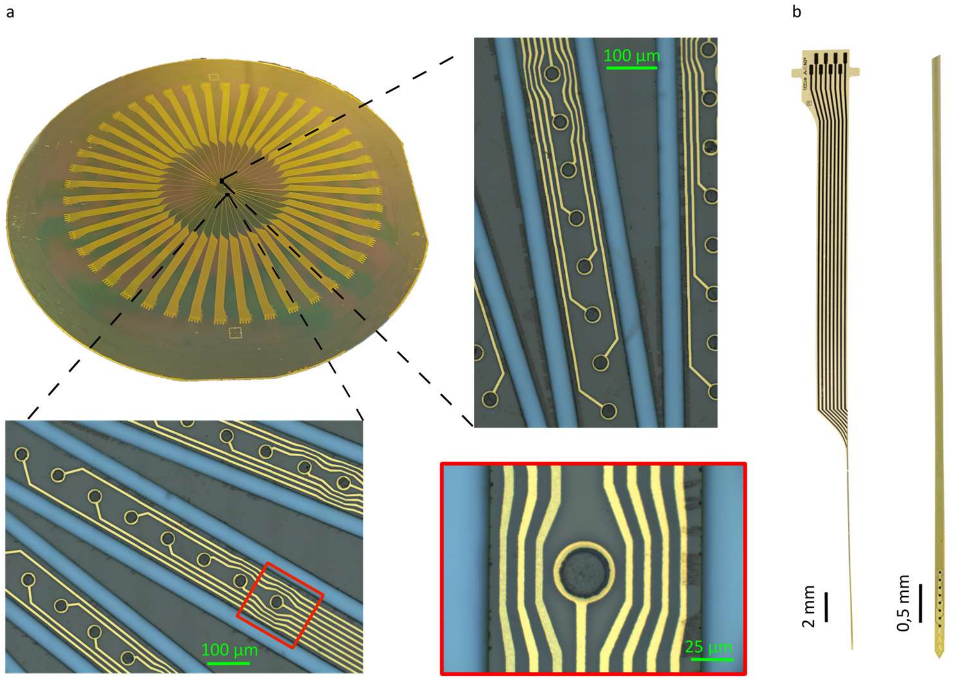

Figure S1 | Flexible thin film microfabrication on a 4-inch Wafer. a, Photograph of a 4-inch wafer with 44 devices microfabricated. 2 zooms of the central part allow for visualization of the tips of the leads where the electrodes are placed. With the red square, a zoom of a single rGO electrode. **b**, Photographs of a device peeled off from the wafer with a zoom of the thin tip.

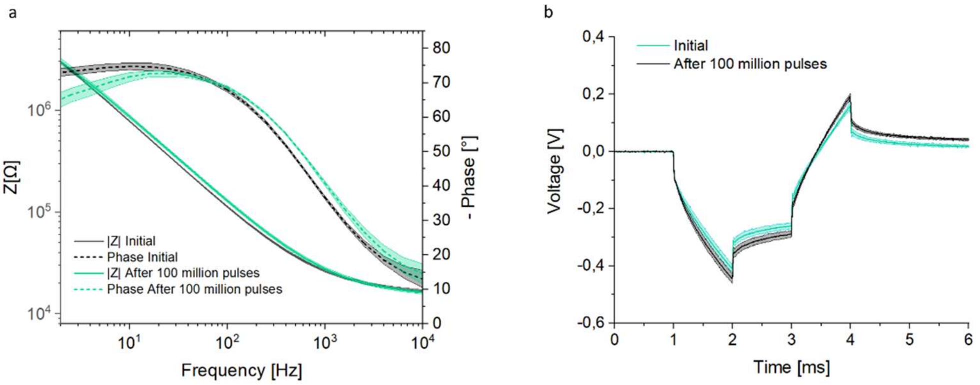

Figure S2 | Stability after long-term stimulation. a, Average impedance magnitude with STD (shadow area), and phase (for 3 electrodes) measured before and after injecting 100 million stimulation pulses (biphasic current pulses of 100 µs in pulse width and 50 µA amplitude). **b,** Average voltage polarization with STD (shadow area) in response to a biphasic current pulse of 1 ms in pulse width and 5 µA amplitude; the measurement is performed (with 3 electrodes) before and after the injection of 100 million pulses with the same protocol of subpanel a.

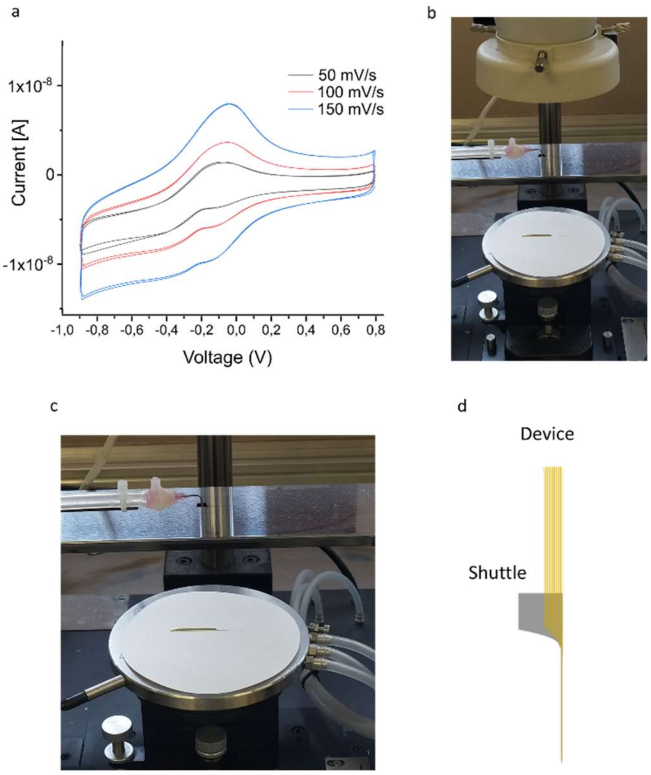

Figure S3 | Electrochemical characterization and device attachment. a, Cyclic voltammograms for one electrode were recorded at different scan rates, from -0.9 V to 0.8 V vs Ag/AgCl, in PBS. **b**,, Set-up used to glue with the adhesive polymer the flexible device to the shuttle. The device is placed on a probe station that can be moved with manipulators. A vacuum pump connects with a plastic tube a metallic tip, that can hold the shuttle. After applying the adhesive polymer with a brash on the tip of the shuttle, the flexible device is aligned with the shuttle looking under the microscope, and then assembled. **d,** Schematic of a device attached to the shuttle.

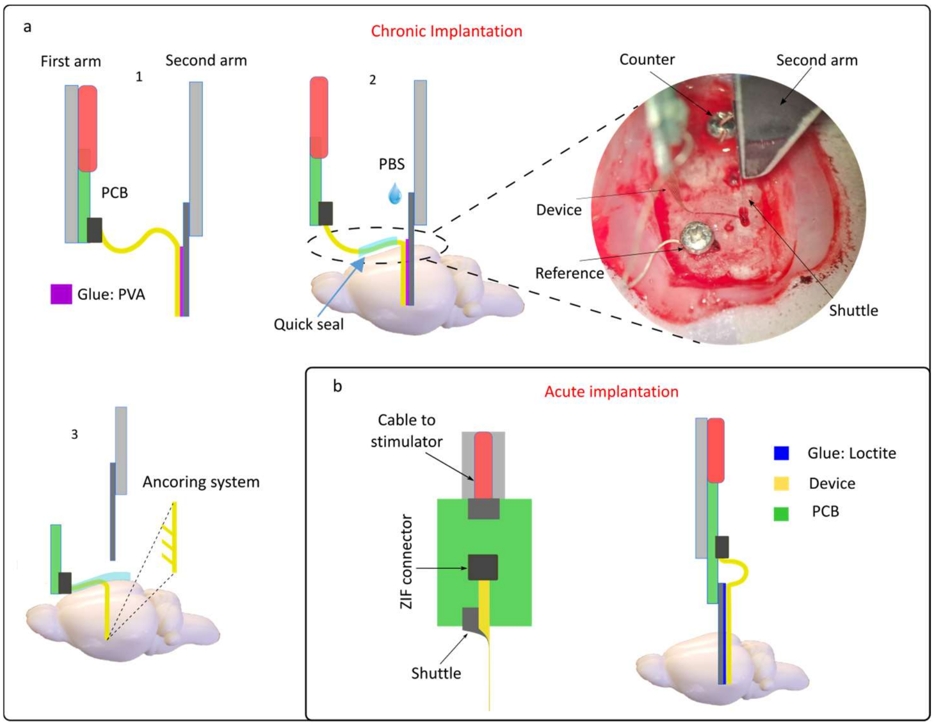

Figure S4 | Insertion strategy in chronic and acute. a, Chronic implantation with a 2 arms strategy in 3 steps. 1 The first arm holds the PCB that connects the tail of the lead, while the second one holds the shuttle attached to the array. 2 The second arm is slowly lowered with a micro driver to insert the device into the brain. When the insertion is completed, the tail is encapsulated with Kwik seal avoiding the insertion hall. PBS is then applied to dissolve the PVA that doesn't contact the brain fluids. The wires of the reference, ground and motor cortex connected to the PCB, are fixed on the scalp using small screws. 3 After 20 min, when the adhesive polymer is dissolved, the shuttle is removed and the device is kept in place thanks to the anchoring system. Everything is then encapsulated. **b**, Acute implantation with one arm insertion strategy; The shuttle is fixed in a free space of the PCB below the ZIF connector. The PCB is then connected to the stimulator and the cable is fixed on a stereotactic arm controlled by the micro driver. The device, glued with a permanent adhesive polymer, is slowly inserted into the brain until reaching the STN.

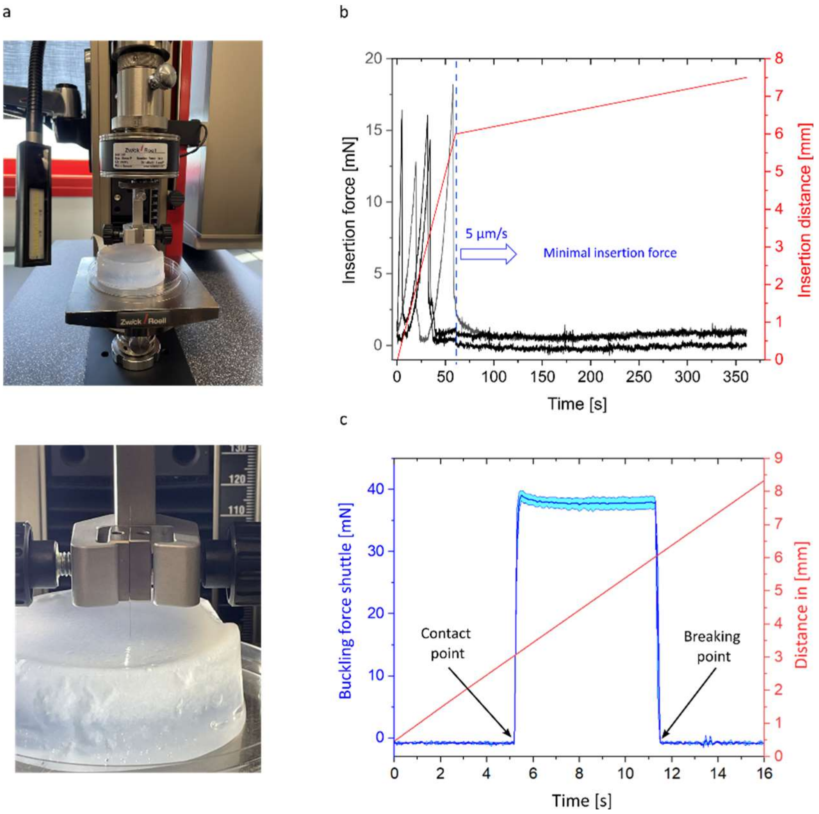

Figure S5 | In vitro test of the insertion force. a, Set-up for the measurement of the insertion force in the agar of a device glued with the shuttle. The device is inserted with the micro driver while a load force cell measures the force produced over time and the displacement. **b,** Insertion force (black) over time in agar for 3 devices glued with the shuttle, following the insertion speed used in vivo, 6 mm at 100 µm/s and 1.5 mm at 5 µm/s. Displacement over time in red. **c,** Average buckling force with STD (shadow area) for 3 shuttles on a rigid surface. The first point around 5 s represents the moment when the shuttle touches the rigid surface. Then it starts to bend under a force of around 37 mN, until the breaking point. The displacement is at the constant speed of 100 µm/s.

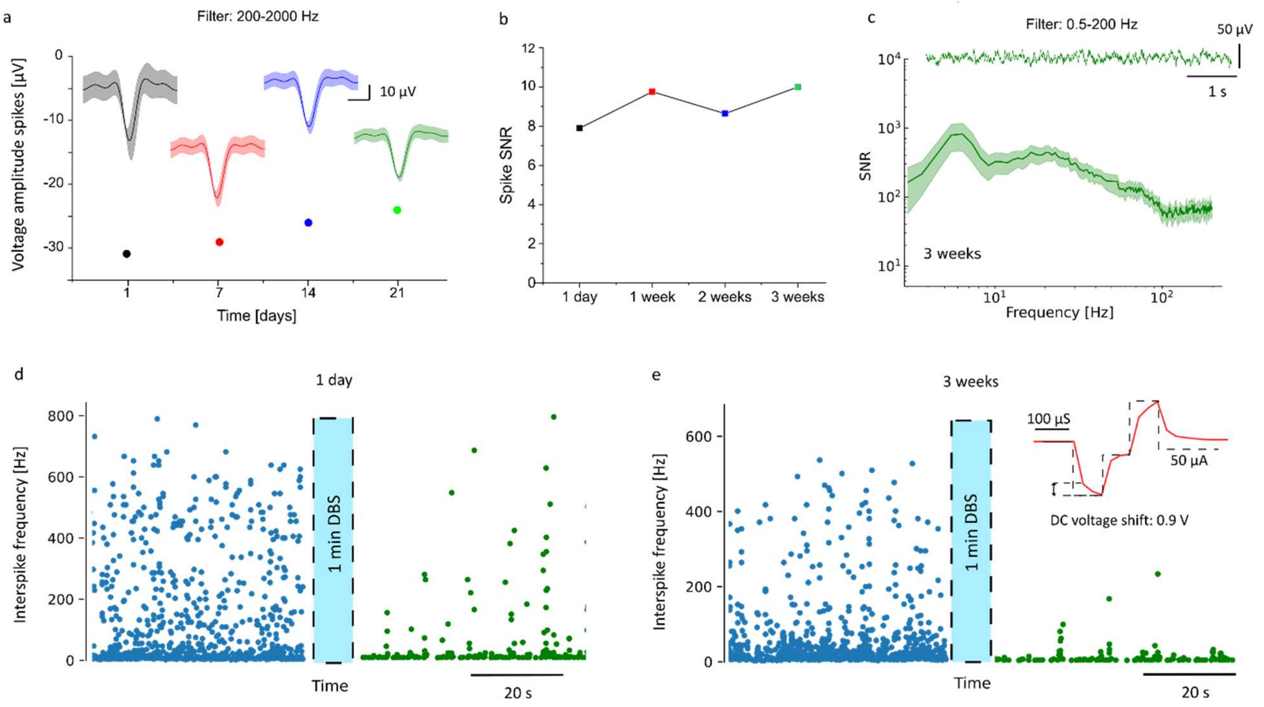

Figure S6 | Proof-of-concept in vivo chronic study of recording and stimulation capabilities. **a**, the spike amplitude was recorded over 3 weeks with one electrode in the STN. On top, the average voltage spike shapes with shadow area for the STD (over 100 s), are extracted at the corresponding time points. **b**, Spike to noise ratio over time of the experiment in the subpanel a. **c,** on top the LFP recording at the third week with one electrode. Below is the average SNR spectrum with STD in the LFP range for the 8 electrodes of the array. **d**, **e**, IF distribution over

time pre- (blue) and post-DBS (green), one day after implantation and after 3 weeks. In red, the measurement shows the electrode voltage polarization measured in the brain during the DBS protocol (biphasic pulses of 50 µA and 100 µs pulse width).

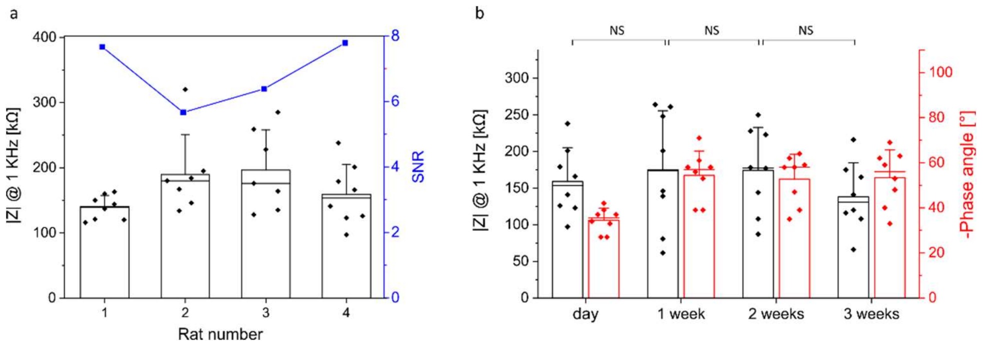

Figure S7 | Electrodes in vivo chronic stability. a, Average impedance magnitude in black (with median line and STD at the whisker) at 1 kHz at day one of 4 arrays implanted in vivo (for each array n=8), with the average spike-to-noise ratio in blue (over 100 s) of one recording electrode in the STN of each implant. **b**, Average impedance magnitude in black and phase in red with median line and STD at the whisker for the 8 electrodes of one array measured in a chronically implanted rat over weeks.

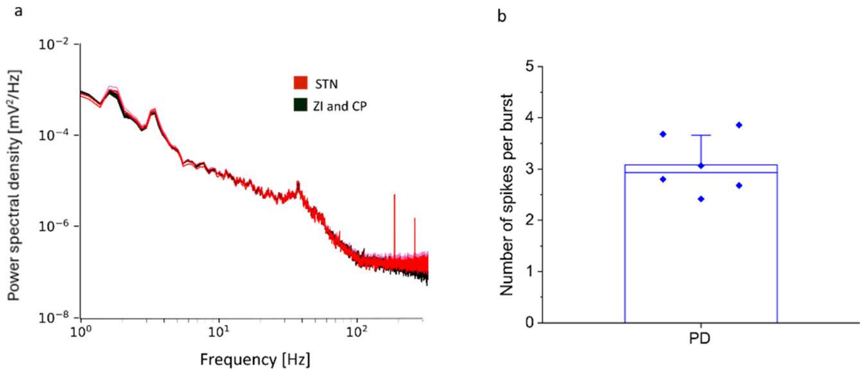

Figure S8| Power spectral density and burst length. a, PSD through the electrodes of one array implanted in the STN. The red one represents the 2 electrodes in the STN while the black ones are placed in the Zona Incerta (ZI) and internal capsule (CP). **b**, Spike to noise ratio over time in the STN of one rat chronically implanted. **b**, Average number of spikes per burst (with median line and STD at the whisker) recorded over 100 s in the STN of the PD group (n=6).

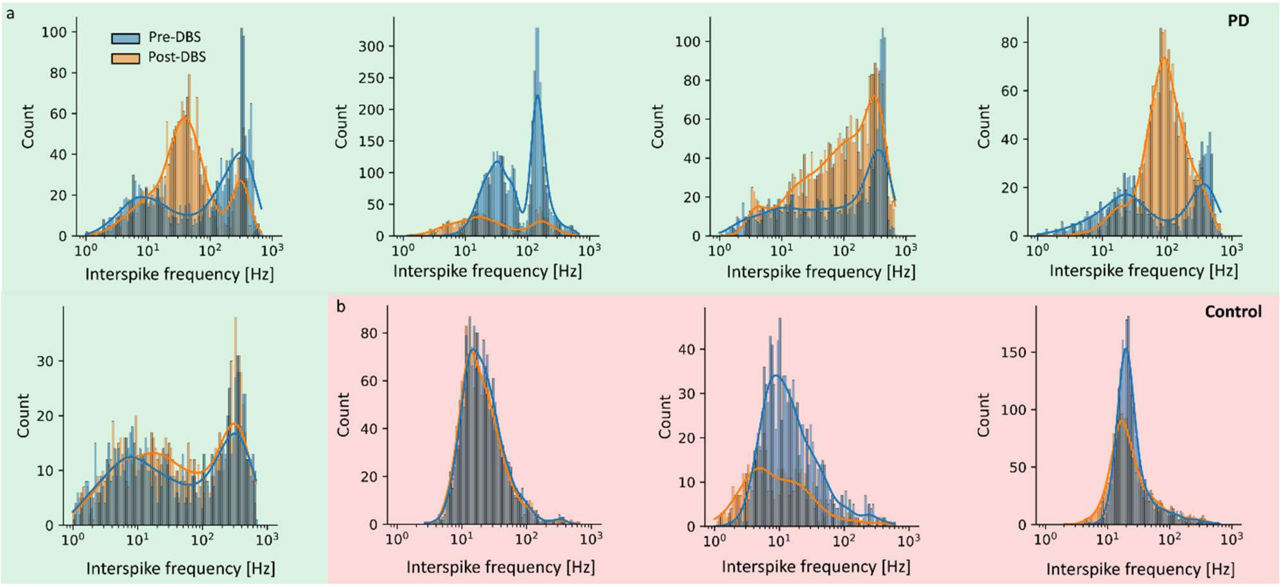

Figure S9 | Spike distribution in the PD-Control. a,b IF distribution pre- (blue) and post-DBS (orange) for the spikes recorded in the STN of 5 PD (green rectangle) and 3 control rats (red rectangle). The histograms are fitted with the kernel density probability.

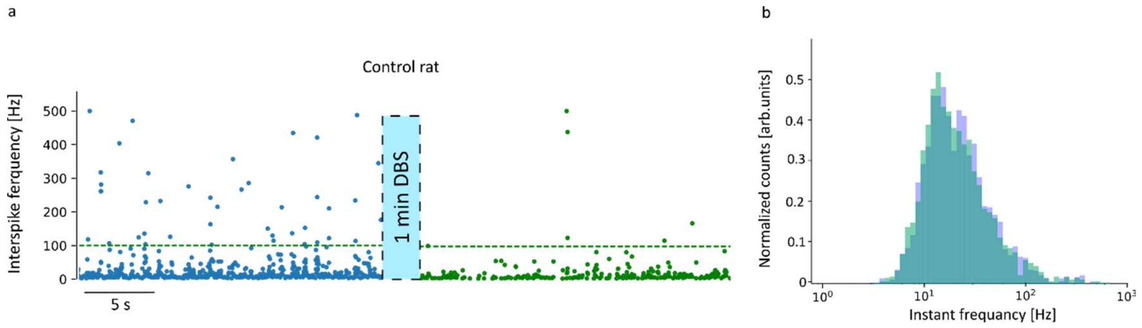

Figure S10 | Effects of DBS on control rat. a, Interspike frequency in the STN of a control rat, pre (blue) and post (green) DBS (75 µA current amplitude, 100 µs pulse width, and 130 Hz frequency). The green dash line highlights the cut-off frequency of the IF for the burst and tonic events. **b**, Normalized interspike frequency distribution over 100 s for the same control rat, pre and post-DBS.

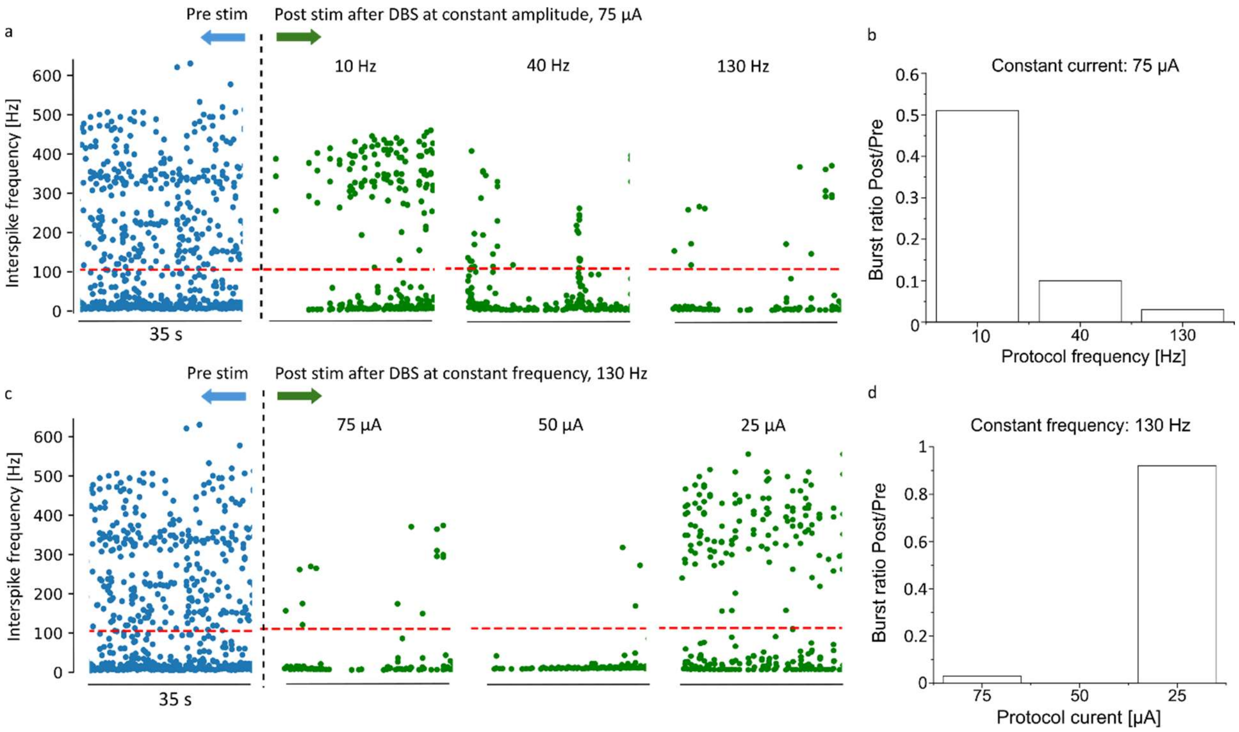

Figure S11 | Effects of DBS parameters on STN spiking activity. **a**, IF over time in the STN of a PD rat in period pre- (blue) and post-DBS (green), for biphasic current pulses of 100 µs and an amplitude of 75uA. Different stimulation frequencies were tested, 10Hz, 40Hz and 130 Hz. The red dash line highlights the cut-off frequency usually used to distinguish burst and tonic IF. **b,** Quantification over 35 s of the burst ratio post/pre for the 3 DBS protocols. **c**, The same effects are evaluated at a fixed frequency (130 Hz) and variable current (25, 50 and 75 µA). **d**, Quantification over 35 s of the burst ratio post/pre for the 3 DBS protocols of variable current.

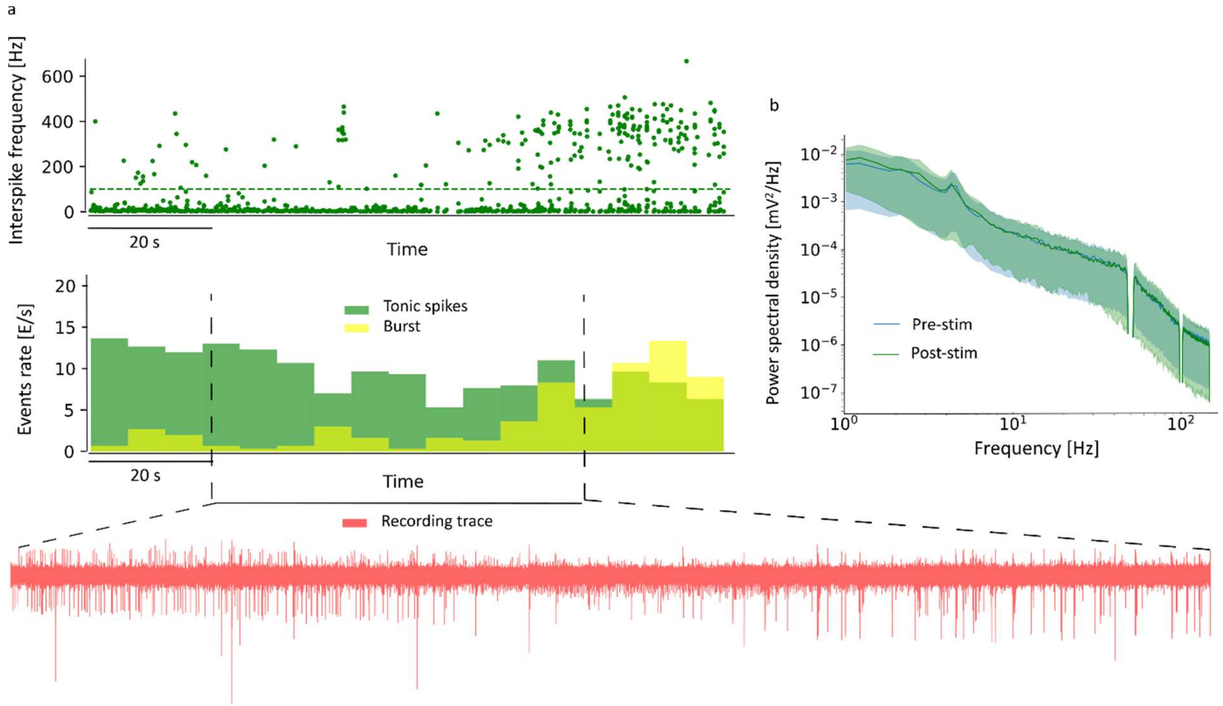

Figure S12 | STN Spiking activity evolution post-DBS. **a,** IF over time in the STN of a PD rat several minutes after DBS (biphasic current pulses of 100 µs, 75uA at 130 Hz). The histogram below quantifies the burst rate (yellow) and tonic spike rate (green) in the same period in 6 s bins. Below is the corresponding zoom of the recording between the 2 dashed lines in the spike range (200-2000 Hz) to visualize the switch from tonic to burst activity. **b**, Average PSD with STD (shadow area) for the recording over 55 s with an electrode in the STD of the PD group (n=6), pre-DBS in blue and post-DBS in green.
