# Supplementary material for: Flexible graphene-based neurotechnology for high-precision deep brain mapping and neuromodulation in Parkinsonian rats
Source: Nat Commun. 2025 Mar 25;16:2891. doi: 10.1038/s41467-025-58156-z (PMC11937542; doi:10.1038/s41467-025-58156-z)
Supplement: Supplementary file 2 — Reporting Summary [file 41467_2025_58156_MOESM2_ESM.pdf]

nature portfolio

Corresponding author(s): DBPR NCOMMS-24-60041A

Last updated by author(s): 18/02/25

Reporting Summary

Nature Portfolio wishes to improve the reproducibility of the work that we publish. This form provides structure for consistency and transparency in reporting. For further information on Nature Portfolio policies, see our Editorial Policies and the Editorial Policy Checklist.

Statistics

For all statistical analyses, confirm that the following items are present in the figure legend, table legend, main text, or Methods section.

n/a	Confirmed	
☐	☒	The exact sample size (*n*) for each experimental group/condition, given as a discrete number and unit of measurement
☐	☒	A statement on whether measurements were taken from distinct samples or whether the same sample was measured repeatedly
☐	☒	The statistical test(s) used AND whether they are one- or two-sided<br>*Only common tests should be described solely by name; describe more complex techniques in the Methods section.*
☒	☐	A description of all covariates tested
☐	☒	A description of any assumptions or corrections, such as tests of normality and adjustment for multiple comparisons
☐	☒	A full description of the statistical parameters including central tendency (e.g. means) or other basic estimates (e.g. regression coefficient) AND variation (e.g. standard deviation) or associated estimates of uncertainty (e.g. confidence intervals)
☐	☒	For null hypothesis testing, the test statistic (e.g. *F*, *t*, *r*) with confidence intervals, effect sizes, degrees of freedom and *P* value noted<br>*Give P values as exact values whenever suitable.*
☒	☐	For Bayesian analysis, information on the choice of priors and Markov chain Monte Carlo settings
☒	☐	For hierarchical and complex designs, identification of the appropriate level for tests and full reporting of outcomes
☒	☐	Estimates of effect sizes (e.g. Cohen's *d*, Pearson's *r*), indicating how they were calculated

Our web collection on statistics for biologists contains articles on many of the points above.

Software and code

Policy information about availability of computer code

Data collection	Standard procedures were used for data collection as especified in the main text.
Data analysis	No custom code was used for data collection. Custom code developed for neurophysiological analysis is available at https://doi.org/10.34810/data1816.

For manuscripts utilizing custom algorithms or software that are central to the research but not yet described in published literature, software must be made available to editors and reviewers. We strongly encourage code deposition in a community repository (e.g. GitHub). See the Nature Portfolio guidelines for submitting code & software for further information.

Data

Policy information about availability of data

All manuscripts must include a data availability statement. This statement should provide the following information, where applicable:

- Accession codes, unique identifiers, or web links for publicly available datasets
- A description of any restrictions on data availability
- For clinical datasets or third party data, please ensure that the statement adheres to our policy

The data and the custom code developed for the analysis of the figures in the main text are available at https://doi.org/10.34810/data1816. Supplementary experimental data that support the figures within this paper and other findings of this study can be accessed by contacting the corresponding authors. Authors can make data available on request, agreeing on the data formats needed.

Research involving human participants, their data, or biological material

Policy information about studies with human participants or human data. See also policy information about sex, gender (identity/presentation), and sexual orientation and race, ethnicity and racism.

Reporting on sex and gender	No human data was used in this project.
Reporting on race, ethnicity, or other socially relevant groupings	No human data was used in this project.
Population characteristics	No human data was used in this project.
Recruitment	No human data was used in this project.
Ethics oversight	No human data was used in this project.

Note that full information on the approval of the study protocol must also be provided in the manuscript.

Field-specific reporting

Please select the one below that is the best fit for your research. If you are not sure, read the appropriate sections before making your selection.

☒ Life sciences ☐ Behavioural & social sciences ☐ Ecological, evolutionary & environmental sciences

For a reference copy of the document with all sections, see nature.com/documents/nr-reporting-summary-flat.pdf

Life sciences study design

All studies must disclose on these points even when the disclosure is negative.

Sample size	This was a technical study which didn't require power size evaluation, since the main part of the experiments were used for methodology validation. 15 rats were used for striatal fluorescence intensity following TH staining. 10 of them were used for electrophysiology study to evaluate recording capability and the detection and modulation of biomarkers. Another additional group of 4 rats, was used to validate the insertion strategy of the lead in the brain and the stability of the electrodes over time.<br>10 devices were used for the electrochemical characterization of the electrodes.
Data exclusions	The rats that during the behavioural test didn't express the proper hemi-parkinsonian behaviour were cosidered not suitable for the implantation of the lead. Experiments where it was not possible to implant properly the device in the targeted brain region, or that present high level of noise due to problems of the electrodes or in the electronic systems, were not included in the data analysis.
Replication	All major findings were replicated (see sample size on figure captions), and the results reproducible. Experimental results were comparable across rats, and electrodes. Single proof-of-concept experiments are also included as demonstration of technological capabilities.
Randomization	Randomization is not rellevant in our study since no conclusion is based on subject-dependent effects.
Blinding	Data analysis was performed by several authors independently and the results converged.

Reporting for specific materials, systems and methods

We require information from authors about some types of materials, experimental systems and methods used in many studies. Here, indicate whether each material, system or method listed is relevant to your study. If you are not sure if a list item applies to your research, read the appropriate section before selecting a response.

Materials & experimental systems

n/a	Involved in the study	
☐	☒	Antibodies
☒	☐	Eukaryotic cell lines
☒	☐	Palaeontology and archaeology
☐	☒	Animals and other organisms
☒	☐	Clinical data
☒	☐	Dual use research of concern
☒	☐	Plants

Methods

n/a	Involved in the study	
☒	☐	ChIP-seq
☒	☐	Flow cytometry
☒	☐	MRI-based neuroimaging

Antibodies

Antibodies used	Anti-TH antibody (AB152; 1:100, Millipore) was used as a primary antibody and alexa fluor anti-rabbit donkey (Alexa Fluoro 647, 1:300, Code A31573) was used as a secondary antibody.
Validation	Antibody concentrations were used based on empirical results from the lab and information provided in the datasheets.

Animals and other research organisms

Policy information about studies involving animals; ARRIVE guidelines recommended for reporting animal research, and Sex and Gender in Research

Laboratory animals	10 Sprague Dawley rats (250–400 g, 8 weeks old) were used for acute electrophysiological recordings and another group of 4 rats to validate the insertion strategy and the chronic stability of the devices in vivo.
Wild animals	none used
Reporting on sex	For the evaluation of the biomarkers in the acute studies, 5 female and 1 male rats were used in the PD group and 2 female and 1 male rats used in the control group . An additional male rat was used to evaluate the recording capability. Data was pooled together for analysis. For the chronic study 4 rats were used. Chronic studies were designed to evaluate the longitudinal performance of the devices and as such sex differences will not influence the results.
Field-collected samples	none used
Ethics oversight	Animal experiments were conducted in accordance with the United Kingdom Animal (Scientific Procedures) Act 1986 and approved by the Home Office (license PPLPP9890301).

Note that full information on the approval of the study protocol must also be provided in the manuscript.

Plants

Seed stocks	No plants were used in this project.
Novel plant genotypes	No plants were used in this project.
Authentication	No plants were used in this project.
